# Sensitivity of Entomopathogenic Fungi and Bacteria to Plants Secondary Metabolites, for an Alternative Control of *Rhipicephalus* (*Boophilus*) *microplus* in Cattle

**DOI:** 10.3389/fphar.2018.00937

**Published:** 2018-08-14

**Authors:** Simona Nardoni, Valentina V. Ebani, Carlo D’Ascenzi, Luisa Pistelli, Francesca Mancianti

**Affiliations:** ^1^Dipartimento di Scienze Veterinarie, Università di Pisa, Pisa, Italy; ^2^Centro Interdipartimentale di Ricerca “Nutraceutica e Alimentazione per la Salute”, Università di Pisa, Pisa, Italy; ^3^Dipartimento di Farmacia, Università di Pisa, Pisa, Italy

**Keywords:** ticks, *Metarhizium anisopliae*, *Beauveria bassiana*, Bacillus thuringiensis, Proteus mirabilis, essential oils, acaricide activity, repellent activity

## Abstract

*Boophilus* (*Rhipicephalus*) *microplus* is a one host hard tick widespread in warm climates worldwide, responsible for great economic losses. To avoid resistance in ticks population, induced by the repeated administration of conventional acaricides and/or the presence of residues in the environment in meat and in milk, an alternative approach can be achieved using entomopathogenic microorganisms such as fungi and bacteria, or essential oils (EOs). The aim of the present study was to evaluate the *in vitro* sensitivity of *Beauveria bassiana, Metarhizium anisopliae*, *Scopulariopsis* sp, *Bacillus thuringiensis* and *Proteus mirabilis* to *Eucalyptus globulus, Lavandula hybrida*, *Pelargonium graveolens* EOs and to their main constituents such as lynalool, linalyl-acetate, geraniol, citronellol and 1,8 cineole. EOs has been chemically characterized by GC-MS. Fungal isolates were tested by a microdilution assay to achieve minimal inhibitory concentration (MIC) of both EOs and main components. The sensitivity of bacteria was evaluated by an agar disk diffusion. The results obtained show the feasibility of an integrate approach for an eco-friendly control of *R. microplus* by use of both entomopathogenic fungi and *P. graveolens* EO. *L. hybrida* could be an interesting alternative when *B. bassiana* is not employed. Conversely, a combined use of *B. thuringiensis* and EOs would not be advisable in the integrate control of ticks.

## Introduction

*Boophilus microplus*, recently recognized as *Rhipicephalus* (*Boophilus*) *microplus*, is a one host hard tick, present in warm climates worldwide. This Ixodidae species is considered the most damaging cattle ectoparasite, acting as vector of *Babesia bigemina, Babesia bovis* and *Anaplasma* *marginale* (Banumathi et al., 2017). Furthermore *R. microplus* can cause anemia, weight loss, and reduction of productive performances (Jonsson, 2006), being together with costs of specific drugs, responsible for great economic losses (Rachinsky et al., 2008).

The control of this agent represents a main concern and relies on both chemical and non-chemical treatments. Conventional control is based on chemical acaricides (Cruz et al., 2015), however repeated administrations of these compounds may induce resistance in tick population (Klafke et al., 2017). Moreover these drugs contaminate the environment as well as meat and, in dairy cows, milk. An alternative approach can be achieved using entomopathogenic microorganisms such as fungi and bacteria, or using active compounds from different botanical species.

Some entomopathogenic fungi, such as *Beauveria bassiana* and *Metarhizium anisopliae*, have been evaluated for ticks’ control (Kaaya et al., 2011; Ren et al., 2016; González et al., 2016; Aw and Hue, 2017). Conversely, *Scopulariopsis brevicaulis*, an environmental deuteromycetes, is considered as a commensal of ticks (Yoder et al., 2005) and would protect some Ixodida species from *M. anisopliae* proliferation (Yoder et al., 2008).

Entomopathogenic bacteria and their derived products are also considered useful for a biological control of arthropods. Some bacterial species have been demonstrated to be pathogenic for ticks. Among them *Bacillus thuringiensis* is the most studied entomopathogen, active versus ticks (Fernández-Ruvalcaba et al., 2010) and largely employed in commercial insecticide formulations. The pathogenic action of this bacterium normally occurs after ingestion of spores and crystalline inclusions containing insecticidal δ-endotoxins that specifically interact with receptors in the insect midgut epithelial cells (Bravo et al., 2007).

Tick pathogenic property of *Proteus mirabilis* has been reported (Brown et al., 1970), also. *P. mirabilis* is an opportunistic bacterium spread in the environment. In fact, it is normally present in the intestinal tract of several animal species, including cattle (Drzewiecka, 2016).

Essential oils (EOs) are secondary plants metabolites, which may show antimicrobial properties (Salehi et al., 2018; Sharifi-Rad et al., 2018a,b,c; Prakash Mishra et al., 2018). Some of them are proven to exert an acaricidal activity (Pirali-Kheirabadi and Teixeira da Silva, 2010, 2011; de Souza Chagas et al., 2012; Banumathi et al., 2017; Rosado-Aguilar et al., 2017). Moreover several EOs posses antifungal and antibacterial activities. So, in view of a combined use of both entomopathogenic organisms and EOs in an eco-friendly way of ticks’ control, the aim of the present study was to evaluate the *in vitro* sensitivity of *B. bassiana, M. anisopliae*, *S. brevicaulis*, *B. thuringiensis* and *P. mirabilis* isolates to *Eucalyptus globulus, Lavandula hybrida* and *Pelargonium graveolens* EOs and to their main constituents. These EOs, in fact would exert both acaricidal and/or repellent activities, respectively, against several tick species. The results obtained would allow the determination of compound concentrations able to exert acaricide and/or repellent actions, without damaging entomopathogenic organisms.

## Materials and Methods

### Essential Oils and Their Major Components

The study was performed employing the following EOs: lavender (*L. hybrida*), eucalyptus (*E. globulus*) and geranium (*P. graveolens*) and some of their major components (lynalool, linalyl-acetate, geraniol, citronellol and 1,8 cineole). All EOs were obtained from the producer (FLORA^®^, Pisa, Italy), while single components were purchased from Sigma (Sigma Aldrich, Germany). They were maintained at 4°C in dark glass vials and were microbiologically analyzed for quality control before use.

#### Gas Chromatography – Mass Spectrometry Analysis

Essential oils were chemically characterized by Gas Chromatography – Mass Spectrometry Analysis (GC-MS). The analysis was performed as previously described (Ebani et al., 2016). Briefly, The GC analysis were accomplished with an HP-5890 Series II instrument equipped with a HP-Wax and HP-5 capillary columns (both 30 m × 0.25 mm, 0.25 μm film thickness), working with the following temperature program: 60°C for 10 min, rising at 5°C/min to 220°C. The injector and detector temperatures were maintained at 250°C; carrier gas, nitrogen (2 mL/min); detector, dual FID; split ratio 1:30. The volume injected was 0.5 μL. The relative proportions of the oil constituents were percentages obtained by FID peak-area normalization without the use of a response factor. GC-MS analyses were performed with a Varian CP-3800 gas chromatograph equipped with a DB-5 capillary column (30 m × 0.25; coating thickness, 0.25 μm) and a Varian Saturn 2000 ion trap mass detector. Analytical conditions were as follows: injector and transfer line temperatures, 220 and 240°C at 3°C/min, respectively; oven temperature, programmed from 60 to 240°C at 3°C/min; carrier gas, helium at 1 mL/min; injection, 0.2 μL (10% hexane solution); split ratio, 1:30. Identification of the constituents was based on comparison of the retention times with those of authentic samples, comparing their linear retention indices relative to the series of n-hydrocarbons, and on computer matching against commercial and home-made library mass spectra built up from pure substances and components of known oils and MS literature data.

### Acaricidal/Repellent Activities of Compounds

Acaricidal activity of selected EOs and both acaricidal and repellent effect of their main components were assayed on *R. microplus* adult engorged females and larvae, respectively, as described by Pirali-Kheirabadi and Teixeira da Silva (2010) and by Wanzala et al. (2014), with slight modification. In detail, serial EOs and components concentrations (0.5, 1, 1.5, 2.0, 3.0, 4.0 and 5 v/v percentages, using 60% ethanol as solvent) were achieved and acaricidal activity was evaluated dipping 5 engorged female ticks. A control group consisted of 5 *R. microplus* adult engorged females, dipped in 60% ethanol without EOs. All determinations were performed in triplicate. Ticks were checked for viability at 6, 18 and 24 h post dipping. Repellent effect was evaluated by tick climbing bioassay (Wanzala et al., 2004), carried out in 5 replicates, using serial components concentrations (0.1, 0.5, 1, 2, 4, 5, 7, 10 v/v percentages) on larvae. The repellency of each concentrations was empirically evaluated counting the number of ticks that climbed the treated and control glass tube respectively.

### Antifungal Activity

#### Fungal Strains

A strain each of 2 entomopathogenic fungi (*M. anisopliae* CBS 115995 and *B. bassiana* CBS 100544) were provided by Westerdijk Fungal Biodiversity Institute (Utrecht, The Netherlands) while an isolate of *S. brevicaulis* was obtained from animal fur specimens; all molds were used for *in vitro* sensitivity assays. All fungi were maintained on Malt Extract Agar (MEA) at room temperature until use.

#### Microdilution Test

The antimycotic activity of EOs was checked by a microdilution test carried out as recommended by Clinical and Laboratory Standards Institute M38-A2 for molds (CLSI, 2008), starting from a 5% dilution. In detail 5, 2.5, 1.5, 1, 0.5, 0.25, and 0.1% dilutions were prepared. Inocula formed by 10^4^ CFU/ml in RPMI were incubated at 25°C evaluated for growth inhibition in 96 - wells plates. The antimycotic activity of all components was evaluated at concentrations of 5, 2.5, 1, 0.5, and 0.25%. MIC was determined as the lowest concentration of each EO that substantially inhibited fungal growth as detected visually. All assays were performed in triplicate.

### Antibacterial Activity

#### Bacterial Strains

*Bacillus thuringiensis* ATCC^®^ 33679^TM^ and *Proteus mirabilis* ATCC^®^ 7002^TM^ were provided by Microbiologics (St. Cloud, MN, United States). Both strains were cultured on Tryptic Soy Broth Agar (Oxoid LTD Basingstoke, Hampshire, England) and incubated at 37°C before the *in vitro* sensitivity test.

#### Minimum Inhibitory Assay

Antibacterial activity and minimum inhibitory concentration (MIC) of each EO and of selected components against both bacterial strains were tested by the broth microdilution method, on the basis of the protocol reported by Lević et al. (2011) with some modification. The bacterial inoculates were prepared using overnight cultures and suspensions were adjusted to 0.5 McFarland standard turbidity. The assay was carried out in Brain Hearth Infusion Broth (BHIB, Oxoid). The test was performed in a total volume of 200 μl/well containing 20 μl of each bacterial suspension, 160 μl of BHIB and 20 μl of each EO/component with final EOs and components concentrations of 10, 5, 2.5, 1.25, and 0.6% (v/v). Plates were incubated at 37°C for 24 h.

The assay was simultaneously executed for sterility control (tested oil/component and BHIB) and bacterial growth control (tested bacteria and BHIB). The MIC value was defined as the lowest concentration of EO/component at which microorganisms show no visible growth. All tests were performed in triplicate.

## Results

### Gas Chromatography – Mass Spectrometry Analysis

The chemical composition of selected EOs is reported in **Table 1**. All the EOs showed high percentages of oxygenate monoterpenes, ranging from 83.4% (*P. graveolens*) to 90.5% (*E. globulus*). *P. graveolens* contained more sesquiterpene hydrocarbons and oxygenate sesquiterpenes, when compared to the other EOs. *L. hybrida* EO was rich in linalool (31.5%) and linalyl acetate (26.8%), *P. graveolens* contained high amounts of citronellol (44.5%) and geraniol (13.7%), while the major components of *E. globulus* were p-cymene (67.7%) and 1,8 cineole (89.8%).

**Table 1:** Chemical composition of tested EOs.

Component	Class of constituents		EO tested		
		LRI^a)^	*L. h*	*P. g*	*E. g*
	
			Relative abundance (%)		
α-Thujene	MH	930			
α-Pinene	MH	939			2.0
Thuja-2,4(10)-diene	MH	960			
Sabinene	MH	975	0.1		
β-Pinene	MH	979	0.4		
α-Phellandrene	MH	1003			
α-Terpinene	MH	1017			
*p*-Cymene	MH	1025			67.7
*o*-Cymene	MH	1026			
Limonene	MH	1029			
β-Phellandrene	MH	1030			
1,8-Cineole	OM	1031	7.7		89.8
γ-Terpinene	MH	1060	0.1		
Terpinolene	MH	1089	0.5		
Linalool	OM	1097	31.5	3.9	
Camphor	OM	1146	7.3		
Menthone	OM	1153		1.1	
Citronellal	OM	1153			
*iso*-Menthone	OM	1163		3.5	
Menthofuran	OM	1164			
Menthol	OM	1172			
4-Terpineol	OM	1177	4.0		
α-Terpineol	OM	1189	2.1	0.3	
Citronellol	OM	1226		44.5	
Neral	OM	1238		0.2	
Geraniol	OM	1253		13.7	
Linalyl acetate	OM	1257	26.8		
Geranial	OM	1267		0.7	
*(E)*-Cinnamaldehyde	NT	1270			
Citronellyl formate	OM	1274		7.3	
Menthyl acetate	OM	1295			
Eugenol	PP	1359			
β-Caryophyllene	SH	1419	2.2	0.7	
Germacrene D	SH	1485	0.8	0.2	
Eugenyl acetate	PP	1523			
δ-Cadinene	SH	1523		0.7	
τ-Cadinol	OS	1640	0.2		
Unknown				0.7	
**Total Identified (%)**			100.0	99.3	100.0

**Class of compounds^a)^**			***L. h***	***P. g***	***E. g***

Monoterpene hydrocarbons (MH)			6.4		9.1
Oxygenated monoterpenes (OM)			85.0	83.4	90.5
Sesquiterpene hydrocarbons (SH)			5.4	7.8	0.3
Oxygenated sesquiterpenes (OS)			1.3	6.9	0.1
Phenylpropanoides (PP)				1.2	
Non-terpenes (NT)			1.9		


*^a)^ Linear retention indices on the DB5 column. L.h.: Lavandula hybrida; P.g.: Pelargonium graveolens; E.g.: Eucalyptus globulus.*

### Acaricidal/Repellent Activities of Compounds

The selected EOs showed different degrees of acaricidal activities. EO from lavender appeared as the most active, with a MIC of 1%, followed by geranium (1.5%), while a 5% concentration of eucalyptus EO did not affect ticks viability. Among the selected compounds, geraniol was acaricide at 1% dilution. The strongest repellent effect was showed by 1,8 cineole (0.5%), followed by geraniol (1%). Linalool failed to show a repellent activity, as well as linalyl-acetate. All replicates within the same tested concentrations of both EOs and components yielded the same value.

### Antifungal Activity

*Lavandula hybrida* EO showed a MIC of 5% for *M. anisopliae* and 1% for *B. bassiana*. *P. graveolens* and *E. globulus* EOs scored active at 5% against both entomopathogenic fungi; geraniol was effective at 1%. *S. brevicaulis* was resistant to more than 5% EO of *P. graveolens* and 2.5% of the other EOs. All components used for repellency testing yielded MIC values higher or equal to repellent ones.

### Antibacterial Activity

*Pelargonium graveolens* and *L. hybrida* EOs showed a MIC of 1.25% against *B. thuringiensis* and 5% versus *P. mirabilis*. *E. globulus* showed a MIC value of 5% for both bacterial strains.

All the selected components were not effective against *P. mirabilis*. *B. thuringiensis* resulted sensible to geraniol, citronellol and linalool at 10%, whereas linalyl-acetate and 1,8-cineole resulted uneffective at the same concentration.

Bacterial growth was detected when bacteria had been incubated only with BHIB, whereas it was not observed in the sterility control wells.

More detailed data are provided in **Table 2**.

**Table 2:** Biological activities of selected essential oils and components against fungi, bacteria and ticks tested in the study.

	*Lavandula hybrida*	*Pelargonium graveolens*	*Eucalyptus globulus*	Geraniol	1,8 cineole	Linalool	Linalyl acetate	Citronellol
*Metarhizium anisopliae*	5	5	5	1	>1	2.5	5	7
*Beauveria bassiana*	1	5	5	1	0.5	2.5	5	2.5
*Scopulariopsis brevicaulis*	2.5	>5	2.5	1	>1	>5	>5	7
*Bacillus thuringiensis*	1.25	1.25	5	10	ne	10	ne	10
*Proteus mirabilis*	5	5	5	ne	ne	ne	ne	ne
*Rhipicephalus microplus*	1	1.5	>5	1/1^∗^	0.5^∗^	>10^∗^	>10^∗^	7^∗^


*^∗^repellent activity. Values are expressed as percentage. Only compounds with relative abundance (%) >3% have been included in this table.*

## Discussion

Results from the present study represent the first evidence about the acaricidal activity of lavender (*L. hybrida*), eucalyptus (*E. globulus*) and geranium (*P. graveolens*) EOs against *R. microplus* engorged females, and their possible use in synergistic effect with entomopathogenic microorganisms. Findings obtained appeared promising, mainly referred to antifungal results.

The three EOs examined in the present study showed different degrees of bioactivity both versus *R. microplus* and tested molds and bacteria. *E. globulus* EO was the only compound previously assayed against ticks (Pirali-Kheirabadi and Teixeira da Silva, 2010) yielding an acaricide action on 37.5% of ticks, when used at a 5% dilution. In the present study, *P. graveolens* was essayed, instead of *P. roseum* (Pirali-Kheirabadi and Teixeira da Silva, 2010) and showed a good activity at 1.5%, while *L. hybrida*, instead of *Lavandula angustifolia* (Pirali-Kheirabadi and Teixeira da Silva, 2011) was active at 1%. Furthermore, among the main components of selected oils, geraniol showed an acaricide effect at 1%. This finding is in agreement with data referred by Singh et al., 2018, who report a larvicide effect in *R. microplus.*

Data about the repellency of the components agreed with recent literature. Indeed, citronellol 7% resulted repellent (Ferreira et al., 2017) as well as geraniol 1% (Khallaayoune et al., 2009) and 1,8-cineol (Pålsson et al., 2008), while linalool (Tabari et al., 2017) and linalyl-acetate scored uneffective.

*Eucalyptus globulus* EO showed the lowest MIC (2.5%) versus *S. brevicaulis*. This mold has been recognized as an endosymbiotic of *Amblyomma americanum* and *Dermacentor variabilis* (Yoder et al., 2005) and is proven to protect this latter from pathogenic actions of *M. anisopliae* (Yoder et al., 2008). Furthermore, both entomopathogenic fungi examined in the present study resulted resistant to such EO dilution. These data would seem to be of interest if these plant-based acaricides would be applied to control other ticks.

*Metarhizium anisopliae* showed a limited sensitivity against the selected EOs, while *B. bassiana* appeared to be sensitive to 1% of *L. hybrida.* The acaricide concentrations of *L. hybrida* and *P. graveolens* EOs have no effectiveness against *P. mirabilis*, but could be lethal for *B. thuringiensis*.

Whereas *P. mirabilis* is a bacterium naturally widespread in the environment, mainly where domestic and/or wild animals are present, *B. thuringiensis* is frequently used in Integrated Pest Management programs for its well-known insecticidal activity (Lucchi and Benelli, 2018). Even though it is considered active against ticks (Samish et al., 2004), *B. thuringiensis* is usually employed against insects of public health and agricultural importance (Ruiu, 2015). For this reason, the administration of *L. hybrida* and *P. graveolens* EOs as biopesticides should be avoided in environment where *B. thuringiensis*-based biocontrol operations are ongoing.

## Conclusion

Results presented here show the feasibility of an integrated approach merging the use of tick repellents and microbial entomopathogens for the eco-friendly management of *R. microplus.* Notably, *Beauveria bassiana* and *Metarhizium anisopliae* can be successfully used in presence of *P. graveolens* EO-based repellents, while *L. hybrida* could be an interesting repellent alternative when *B. bassiana* is not employed. Conversely, a combined use of *B. thuringiensis* and EOs would not be advisable in the Integrated Vector Management of cattle ticks.

## Author Contributions

VE, SN, and FM conceived and designed the experiments. CD, SN, LP, and VE performed the experiments. VE, SN, LP, and FM analyzed the data and wrote the paper.

## Conflict of Interest Statement

The authors declare that the research was conducted in the absence of any commercial or financial relationships that could be construed as a potential conflict of interest.
